# Targeting lipophagy and nuclear receptors in metabolic-associated fatty liver disease: insights from traditional Chinese medicine

**DOI:** 10.3389/fphar.2025.1744069

**Published:** 2026-02-03

**Authors:** Ting Yue, Nafei Huang, Ziming Zhao, Lili Yu, Xiaoming He, Xiao Yu, Yi Zheng

**Affiliations:** 1 The Second Clinical Medical College of Zhejiang Chinese Medical University, Hangzhou, Zhejiang, China; 2 Faculty of Chinese Medicine, Medical Sciences Division, Macau University of Science and Technology, Macao, China; 3 State Key Laboratory of Mechanism and Quality of Chinese Medicine, Macau University of Science and Technology, Macao, China; 4 Macau University of Science and Technology Zhuhai MUST Science and Technology Research Institute, Zhuhai, Guangdong, China; 5 The Second Affiliated Hospital of Zhejiang Chinese Medical University, Xinhua Hospital of Zhejiang Province, Hangzhou, Zhejiang, China

**Keywords:** lipophagy, liver, MAFLD, nuclear receptors, traditional Chinese medicine

## Abstract

Background: Metabolic associated fatty liver disease (MAFLD) represents a globally prevalent chronic hepatic disorder hallmarked by excessive lipid accumulation within hepatocytes. This condition can progressively deteriorate to cirrhosis and hepatocellular carcinoma, thereby imposing a substantial healthcare burden. Numerous laboratory studies confirm that drugs targeting the nuclear receptor (NR)-lipophagy axis exhibit preventive and therapeutic potential for MAFLD. However, most remain confined to animal and cell models, and no specific MAFLD therapies are clinically available. In contrast, traditional Chinese medicine (TCM) has garnered considerable interest due to its unique theoretical framework and clinical efficacy in MAFLD management. Purpose: This article systematically reviews existing herbal compounds, extracts, and active metabolites that target the NR-lipophagy interaction for MAFLD treatment. It aims to facilitate the development of low-side-effect herbal formulations and offer valuable insights for future research on the NR-lipophagy axis. Method: Search terms including “MAFLD”, “nuclear receptor”, “lipophagy”, “Compound Traditional Chinese Medicine”, “active metabolites”, “natural products”, and “disease” were combined for literature retrieval. Result: As modulators of pleiotropic NRs, certain herbal metabolites mimic endogenous ligands to exert regulatory lipolysis effects—synergistically modulating lipid metabolism, mitigating oxidative stress, and suppressing inflammation during MAFLD intervention. Herbal compound preparations modulate the NR-lipophagy axis *via* multi-target, multi-pathway mechanisms: moderately activating fatty acid (FA) oxidation pathways while repressing lipogenesis, thereby achieving sustained amelioration of MAFLD. Conclusion: TCM (including herbal compound preparations, extracts, and active metabolites) exerts therapeutic effects on MAFLD by inducing lipophagy through diverse pharmacological mechanisms and NR-associated signaling pathways. These agents emerge as promising focal points for MAFLD basic research and potential candidates for MAFLD drug development, offering reduced side effects and enhanced therapeutic efficacy.

## 1 Introduction

Metabolic associated fatty liver disease (MAFLD) is a type of fatty liver disease related to systemic metabolic disorders. Studies have shown that MAFLD has become the most common liver disease worldwide, affecting over 30% of the population (Ayada et al., 2022). The disease can progress to steatohepatitis、cirrhosis and/or liver cancer (Alqahtani et al., 2023; Wong et al., 2023; Fouad et al., 2024), seriously affecting the quality of life of patients. Regarding its pathogenesis, dysfunction of adipose tissue along with related insulin resistance and low-grade inflammatory responses, can lead to increased synthesis of triglycerides (TGs) in the liver and reduced metabolism. These factors cause liver inflammatory damage and stellate cell activation through mechanisms such as mitochondrial dysfunction and endoplasmic reticulum stress, thereby causing MAFLD (Eslam et al., 2020; Loomba et al., 2021).

Lipophagy selectively degrades lipid droplets (LDs) *via* the autophagolysosomal pathway, yielding free fatty acids (FFAs) (Singh et al., 2009). Currently, this autophagy-lipolysis-dependent pathway for LDs degradation is recognized as crucial in sustaining the homeostasis of hepatic lipid metabolism (Zhang et al., 2022). Studies indicate that lipophagy inhibitory factors is enhanced in the livers of MAFLD patients, accompanied by impaired lipophagic function (Tanaka et al., 2016). Augmenting lipophagy facilitates the clearance of excess accumulated TGs (Carracedo et al., 2013), thereby alleviating hepatic burden.

NRs are ligand-dependent transcription factors that regulate various biological processes upon ligand activation. Among these, peroxisome proliferator-activated receptors (PPARs) (Wagner and Wagner, 2020), farnesoid X receptor (FXR) (Zheng et al., 2023), and liver X receptor (LXR) (Wang and Tontonoz, 2018; Kim et al., 2023) act as central regulatory hubs in metabolic control, playing pivotal roles in sustaining the holistic balance of energy metabolism and metabolic homeostasis, has been confirmed to be related to MAFLD.

Chinese herbal medicines and their extracts have emerged as promising sources for developing therapeutic agents to prevent and treat MAFLD (Yan et al., 2020). Current research indicates that specific TCM metabolites can interact with NRs, actively and passively regulating physiological responses by modulating NR signaling and their transcriptional networks. These metabolites exert holistic regulatory effects on lipid metabolism, inflammation, and oxidative stress, thereby providing a novel perspective for MAFLD intervention (Li et al., 2015).

## 2 Methods

This paper searched for studies using the keywords “MAFLD”, “nuclear receptor”, “lipophagy”, “Compound Traditional Chinese Medicine”, “active metabolites” “natural products” and “disease”. These keywords should be combined in pairs or multiple combinations to enhance the breadth and precision of the literature search. PubMed (https://pubmed.ncbi.nlm.nih.gov), and Web of Science (http://apps.webofknowledge.com/) were selected.

The inclusion criteria for this systematic review encompass three key domains: targeted lipophagy therapies for MAFLD, molecular mechanisms underlying NR-mediated regulation of lipophagy, and studies investigating TCM compound prescriptions or metabolites that target NRs and lipophagy for MAFLD treatment.

All published studies in English, including *in vitro* and *in vivo* experiments, were included without any language restrictions. First, we selected articles based on their titles and then abstracts. Finally, we analyzed the full text in detail, including article source, acquisition route, chemical composition, pharmacological effect, and side effects. We excluded articles with only an indirect relevance to the treatment of MAFLD targeting NRs and lipophagy. Incomplete data, case reports, editorials, posters, and conference abstracts were excluded.

## 3 Lipophagy in metabolic liver disease

### 3.1 Molecular mechanisms

The process of autophagy encompasses five stages as follows (Takahashi et al., 2018).

The mammalian target of rapamycin complex 1 (mTORC1) and AMP-activated protein kinase (AMPK) serve as pivotal regulatory kinases in autophagy (Tamargo-Gómez and Mariño, 2018). During initiation, upon mTORC1 inactivation, autophagic induction hinges on two core complexes: the Unc-51-like autophagy-activating kinase 1 (ULK1) complex and the class III phosphatidylinositol 3-kinase (PI3K) complex. The ULK1 complex comprises ULK1 or ULK2, ATG13, ATG101, and the 200 kDa focal adhesion kinase family-interacting protein (FIP200) (Lee and Jeon, 2020; Xiang et al., 2020).

Subsequently, the process proceeds to phagophore elongation and autophagosome formation. The PI3K complex consists of VPS34, Beclin1, VPS15, ATG14L, and the autophagy/Beclin1 regulatory factor (Activating molecule in BECN1-regulated autophagy protein 1, AMBRA1) (Wirth et al., 2013; Stjepanovic et al., 2017). AMPK induces lipophagy through phosphorylating and activating both the ULK1 and PI3K complexes, mTOR and AMPK collaborate with diverse factors to maintain precise lipophagic activity (Kim et al., 2008). Upon various stimuli, the ULK1 complex activates the PI3K complex, which in turn generates localized phosphatidylinositol 3-phosphate (PI3P) to recruit WD repeat domain phosphoinositide-interacting proteins (WIPIs) and double FYVE-containing protein 1 (DFCP1) (Li and Peng, 2022).

Phagophore nucleation commences with the formation of a PI3P platform. The phagophore then dissociates from the endoplasmic reticulum (ER) and engulfs ER-derived lipid LDs to facilitate nucleation. Following nucleation, the phagophore elongates into an autophagosome *via* two ubiquitin-proteasome systems. The ATG12 system promotes conjugation of ATG8 to phosphatidylethanolamine on autophagic membranes, forming microtubule-associated protein 1A/1B-light chain 3-II (LC3-II). LC3-II on phagophore membranes interacts with ubiquitin-dependent selective autophagy receptors (e.g., P62, OPTN, NBR1) to deliver “cargo” into the phagophore (Rasmussen et al., 2022; Vargas et al., 2023).

Following autophagosome formation, the vesicles undergo docking and fusion with lysosomes. Upregulation of lysosomal gene expression orchestrates lysosome biogenesis and augments autophagic flux. Under starvation conditions, the mucolipin TRP cation channel 2 (MCOLN2) activates dephosphorylates transcription factor EB (TFEB), which translocates to the nucleus, where it binds CLEAR promoter elements within the lysosomal gene network to upregulate lysosomal gene expression (Franco-Juárez et al., 2022). Additionally, transcription factor E3 (TFE3) translocates to the nucleus and binds CLEAR elements, synergistically promoting lysosomal biogenesis (Martina et al., 2014).

Ultimately, within autolysosomes, lysosomal acid lipase (LAL) degrades TGs stored in LDs into FFAs, which are then released into the cytoplasm. LAL expression is transcriptionally governed by factors such as PPARs, FoxO1, TFEB, and TFE3. Rab32 and FoxO1 promote LD breakdown, while CCND1 inhibits this process; DNM2 facilitates both FFAs release and the generation of new lysosomes (Vargas et al., 2023).

### 3.2 Lipophagy in MAFLD

The dysregulation of lipophagy disrupts lipid homeostasis, leading to aberrant lipid accumulation. Studies reveal that FFAs derived from adipose tissue and hepatic *de novo* lipogenesis (DNL) serve as primary sources of TG accumulation in MAFLD (Donnelly et al., 2005), impaired autophagy correlates with increased susceptibility to MAFLD (Allaire et al., 2019). For instance, Rubicon—a negative regulator of autophagy—is overexpressed in MAFLD patients; conversely, hepatic Rubicon knockout in mice confers protection against MAFLD (Levine and Kroemer, 2019). Similarly, downregulation of the immune-related GTPase family M (IRGM) gene—a key autophagic regulator—triggers autophagic flux stagnation, induces hepatic LD accumulation (Li et al., 2016). In SOD1-deficient mice, hallmark features of impaired lipophagy (elevated LC3-II, aberrant P62 deposition) contribute to liver injury and pathological LDs accumulation (Kurahashi et al., 2015).

Dysregulation of lipophagy-related molecular pathways further exacerbates metabolic imbalance. Smith et al. demonstrated that AMPK activation mitigates MAFLD through coordinated effects: inhibiting hepatic DNL, maximizing hepatic FA oxidation, and enhancing adipose mitochondrial function (Smith et al., 2016). In MAFLD models, silencing sterol regulatory element-binding protein 2 (SREBP-2) restores autophagic flux and upregulates autophagy-related gene expression (Deng et al., 2017). Activation of fibroblast growth factor 21 (FGF21) ameliorates MAFLD phenotypes by promoting autophagy (e.g., LC3-II upregulation, increased autophagic flux), even in overweight mice (Zhu et al., 2016).

### 3.3 Drugs targeting lipophagy

The clinically used drugs for regulating autophagy primarily include rapamycin and metformin. As an mTOR inhibitor, rapamycin forms a rapamycin-FKBP12 complex *in vivo*, which specifically binds to mTORC1 and modulates multiple stages of autophagy. This mechanism involves reducing the phosphorylation of proteins such as ULK1 and ATG13, thereby promoting the initiation and nucleation of autophagy (Shimobayashi and Hall, 2014); it also facilitates the elongation of autophagic membranes by modulating the degradation of WIPI2 and the lipidation of LC3-II (Wan et al., 2018). However, conflicting reports exist regarding the regulation of TFEB/TFE3 phosphorylation and nuclear translocation by rapamycin, and its efficacy is limited in TSC2-positive cells (Peña-Llopis et al., 2011). Clinically, rapamycin is associated with multiple adverse effects, including oral ulcers, rashes, anemia, and hyperglycemia. As an immunosuppressant, it increases susceptibility to infections and may induce complications such as lymphoma and other malignancies (e.g., skin cancer) (Nguyen et al., 2019).

Metformin activates AMPK through two pathways: first, by inhibiting mitochondrial respiratory chain complex I or AMP deaminase to reduce ATP production; second, by directly binding to AMPK subunits and promoting activation of upstream AMPK regulators, thereby activating the AMPK/mTOR pathway to induce lipophagy (Luengo et al., 2014). Furthermore, metformin promotes AMPK activation and hepatic FGF21 expression (Nygaard et al., 2012), FGF21, in turn, activates AMPK *via* multiple pathways to enhance lipophagy FGF21 (Lewis et al., 2019). Metformin primarily inhibits hepatic mitochondrial respiration, increasing plasma lactate levels in a concentration-dependent manner and potentially inducing metformin-associated lactic acidosis, which has a relatively high mortality rate (Zhai et al., 2018).

## 4 NRs as therapeutic targets

### 4.1 NRs in lipid metabolism

NRs constitute a superfamily of ligand-dependent transcription factors that act as intracellular sensors for lipid metabolites and nutrients, transducing cues into transcriptional programs that sustain metabolic homeostasis. They play a pivotal role in orchestrating metabolic and inflammatory balance, regulating lipid metabolism and inflammation through multiple pathways.

As a BA-activated nuclear transcription factor, FXR is highly expressed in the liver, ileum, kidney, and adrenal gland (Parks et al., 1999). In non-alcoholic steatohepatitis (NASH), hepatic FXR activation exerts systemic effects by upregulating FA β-oxidation, suppressing DNL and FA synthesis *via* inhibition of sterol regulatory element-binding protein-1c (SREBP-1c), and reducing cholesterol/triglyceride synthesis and glucose production through enhanced FGF21 secretion—thereby ameliorating insulin resistance. Additionally, FXR activation mitigates inflammatory responses by inhibiting NF-κB, NLRP3, and CCL-2, while attenuating fibrosis *via* suppression of TGF-β1 secretion and extracellular matrix (ECM) deposition by hepatic stellate cells (Adorini and Trauner, 2023).

LXRs, comprising LXRα and LXRβ, are expressed predominantly in the liver (LXRα) or ubiquitously (LXRβ) (Yonezawa et al., 2019). Liver X receptors α and β (LXRα/β) share high structural similarity, with ∼77% sequence homology in their DNA-binding domains (DBDs) and ligand-binding domains (LBDs). LXRα preferentially associates with transcriptional repressors, whereas LXRβ binds more robustly to co-activator peptides (Hu et al., 2003; Buñay et al., 2021). LXRα/β typically share downstream target genes and exhibit compensatory effects in the transcriptional regulation of specific genes. They jointly upregulate SREBP-1c and its targets—fatty acid synthase (FASN), and stearoyl-CoA desaturase-1 (SCD-1), acetyl-CoA carboxylase (ACC)—thereby enhancing hepatic FA synthesis and lipid accumulation (Higuchi et al., 2008). Additionally, LXRα/β induce the expression of ATP-binding cassette transporters A1 and G1 (ABCA1/ABCG1), which facilitate cholesterol efflux from macrophages to high-density lipoprotein (HDL) and apolipoproteins (APOs), driving reverse cholesterol transport (RCT) to the liver and promoting hepatic cholesterol excretion (Bischoff et al., 2010). Collectively, these pleiotropic effects of LXRα/β contribute to the pathogenesis and progression of MAFLD (Han et al., 2019).

PPARs are fatty acid-activated transcription factors that govern energy metabolism, with three subtypes: PPARα, PPARγ, and PPARβ/δ.

PPARα inhibits DNL by downregulating SREBP-1c expression (Francque et al., 2015). It also upregulates APO A1/A2 expression to modulate lipid transport and mitigate hepatic steatosis (Sanderson et al., 2010), and suppresses TGF-β expression—attenuating hepatic stellate cell activation and fibrosis *via* multiple pathways to alleviate MAFLD (Chen et al., 2015). PPARβ/δ inhibits DNL by suppressing SREBP-1c expression (Zarei et al., 2018). It upregulates APO and hepatic glucokinase expression, induces autophagic activation (Tong et al., 2019). Additionally, PPARβ/δ inhibits NF-kB activity, suppresses the production of pro-inflammatory cytokines linked to insulin resistance, and ameliorates MAFLD (Rodríguez-Calvo et al., 2008). PPARγ exerts a bidirectional regulatory role in MAFLD pathogenesis. On one hand, it upregulates the expression of FASN, ACC1, phosphoenolpyruvate carboxykinase, and glycerol kinase—promoting lipid accumulation and potentially exacerbating MAFLD (Lu et al., 2014; Skat-Rørdam et al., 2019). On the other hand, PPARγ enhances macrophage M2 polarization, suppresses the secretion of TNF-α and MCP-1, alleviates inflammation and fibrosis to mitigate MAFLD progression (Skat-Rørdam et al., 2019).

### 4.2 Drugs targeting NRs

Obeticholic acid (OCA), a synthetic derivative of the natural BA chenodeoxycholic acid, acts as a selective FXR agonist. Mudaliare et al. had improved insulin sensitivity, reduced body weight, and significantly decreased serum levels of γ-glutamyl transferase (GGT) and alanine aminotransferase (ALT). Additionally, OCA alleviated hepatic inflammation and fibrosis markers in patients with type 2 diabetes and non-alcoholic fatty liver disease (NAFLD) (Mudaliar et al., 2013). Neuschwander-Tetri et al. further confirmed that OCA ameliorates histological features of NASH, including hepatic steatosis, hepatocellular ballooning, lobular inflammation, and fibrosis (Neuschwander-Tetri et al., 2015). However, clinical studies have reported adverse effects of OCA, including pruritus, dyslipidemia, and increased risk of gallstone formation (Al-Dury et al., 2019; Younossi et al., 2019).

Fibrates (phenoxyisobutyric acid derivatives) function as PPARα agonists. Upon binding to PPARα, fibrates promotes FFAs oxidation, reduces TG levels, and increases HDL cholesterol (HDL-C) levels to exert lipid-lowering effects. Zhang and Hansen’s experiments demonstrated the aforementioned functions of Fibrates and also showed that they could improve liver steatosis (Hansen et al., 2010; Zhang et al., 2015b). Common adverse reactions of fibrates include mild, tolerable gastrointestinal symptoms (e.g., nausea, vomiting, diarrhea). Infrequently, patients may experience transaminitis or myalgia, though the incidence remains low.

### 4.3 TCM and NRs

Herbal medicines and plant extracts are natural resources exhibiting properties such as wide availability, functional diversity, safety, and convenience, with minimal residues effects—rendering them promising candidates for drug development (Veiga et al., 2020). In China, the ancient concept of “food as medicine” has long been recognized, and modern society has witnessed growing attention to the role of herbal medicines and plant extracts in treating metabolic fatty liver diseases (Van De Wier et al., 2017; Frasinariu et al., 2022). Notably, certain phytochemicals have demonstrated efficacy in inhibiting hepatic lipid deposition through interactions with NRs. For instance, Saikosaponin has been shown to activate PPARα. Specifically, by inhibiting FA synthesis and promoting FA β-oxidation, it modulates lipid homeostasis in a favorable manner (Gu et al., 2022). Berberine (BBR) activates the energy-sensing AMPK/SIRT1 axis, increasing PPARγ deacetylation to promote adipose tissue remodeling (Xu et al., 2021). In type 2 diabetic mice, BBR enhances glucose utilization and reduces TG uptake/synthesis by regulating NR expression—specifically increasing LXR and PPAR levels while decreasing SREBP expression in white adipose tissue (Liu et al., 2010). As an LXR antagonist, Rhein significantly upregulates energy metabolism in mice, ameliorating glucose tolerance and insulin resistance, lowering serum cholesterol levels, and reversing hepatic steatosis (Sheng et al., 2011).

## 5 TCM modulates lipophagy *via* NRs

Given the pivotal role of lipophagy in regulating hepatic LDs, NRs modulate multiple facets of lipophagy to govern lipid metabolic processes, thereby influencing the pathogenesis of metabolic diseases such as MAFLD. TCM exhibits therapeutic potential in ameliorating MAFLD by targeting lipophagy through interactions with NR sensors and their transcriptional networks.

### 5.1 Direct regulation

NRs directly regulate the transcription of lipophagy-related genes by binding to their promoter regions. As a master regulator of the autophagy-lysosome pathway, TFEB is recognized as a key controller of autophagy gene expression. Studies have identified an estrogen-related receptor (ERR) response element within the TFEB promoter. Activation by ERR agonists induces TFEB expression in cells, thereby enhancing lysosomal biogenesis, promoting autophagolysosome formation, and improving lipophagic efficiency (Losby et al., 2024).

NRs also modulate autophagy by governing the transcription of autophagy pathway-related genes (Lee et al., 2014). For example, PPARγ agonists activate AMPK through adiponectin signaling and AMPKα phosphorylation (Lee and Kim, 2010). AMPK inhibits the mTORC1 pathway *via* phosphorylation of targets such as tuberous sclerosis complex 2 (TSC2) and Raptor (Inoki et al., 2012). Under mTORC1 inhibition, ULK1-mediated phosphorylation of ATG14 is upregulated, facilitating lipophagosome formation (Park et al., 2016). Additionally, TZDs directly upregulate LC3-II expression *via* the AMPK/mTOR pathway, promoting autophagosome formation and enhancing lipophagy. ATG16L1, a component of the autophagy machinery involved in autophagosome biogenesis, is regulated by the vitamin D receptor (VDR). Upon ligand binding, VDR translocates to the nucleus and binds to promoter regions of target genes (e.g., ATG16L1), thereby regulating autophagy. Reduced VDR expression decreases ATG16L1 levels, inhibiting lipophagosome formation and impairing lipophagic function (Sun, 2016).

**FIGURE 1:**
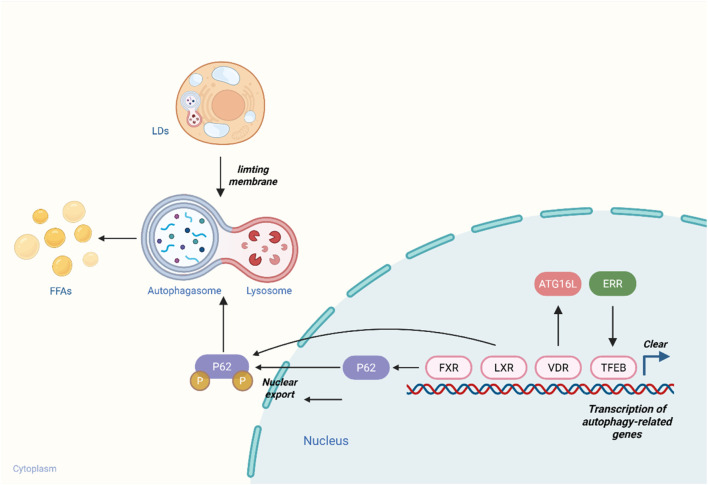
Nuclear Receptors Directly Regulate the Expression of Lipophagy-related Genes. NRs directly regulate transcription by binding to the promoter regions of LD-associated genes. The ERR response element is located in the promoter region of TFEB; ERR binding to this element upregulates TFEB expression, thereby promoting lysosome biogenesis. VDR interacts with ATG16L to induce autophagosome formation. FXR upregulates P62 expression, while LXR induces P62 ubiquitination—together promoting autophagolysosome formation and facilitating the degradation of LDs into free FAs. This figure was created with BioRender.com.

As a lipophagy receptor, P62 recognizes and sorts cytoplasmic cargo *via* its ubiquitin-binding domain, interacting with LC3/ATG8 family proteins to target substrates to autophagosomes. LXR activation induces P62 ubiquitination, while the FXR-specific agonist GW4064 has been shown to upregulate hepatic P62 gene and protein expression in mice, enhance P62-LD interactions, and promote lipophagosomal engulfment of LDs (Haga and Ozaki, 2017) (Figure 1).

### 5.2 Indirect regulation

Within the PPAR family, PPARα forms a heterodimer with retinoid X receptor (RXR) to specifically bind peroxisome proliferator response elements (PPREs) in target gene promoters. This interaction upregulates the expression of key FA oxidases, including CPT-1A and ACOX1, thereby reducing intracellular LD accumulation, and decreasing lipophagic demand to maintain cellular energy homeostasis (Rakhshandehroo et al., 2010). Upon activation, PPARγ promotes LD biogenesis by regulating target gene expression, particularly upregulating FSP27 to facilitate lipid storage in larger, more stable droplets. This sequestration of excess FFAs mitigates lipotoxicity risks. Additionally, the stable LDs structure may inhibit lipophagic degradation of stored lipids, establishing an antagonistic regulatory balance in energy metabolism (Sharma et al., 2019). Under fed conditions, PPARγ induces FGF21, which acts locally in adipose tissue to enhance lipid oxidation and suppress lipogenesis (Ahmadian et al., 2013).

LXR functions as a central regulator of SREBP expression, governing DNL and cholesterol synthesis. The selective LXR agonist T0901317 binds LXR to activate SREBP-1c transcription, subsequently upregulating lipogenic target genes such as FASN and SCD-1 (Beaven et al., 2013).

**FIGURE 2:**
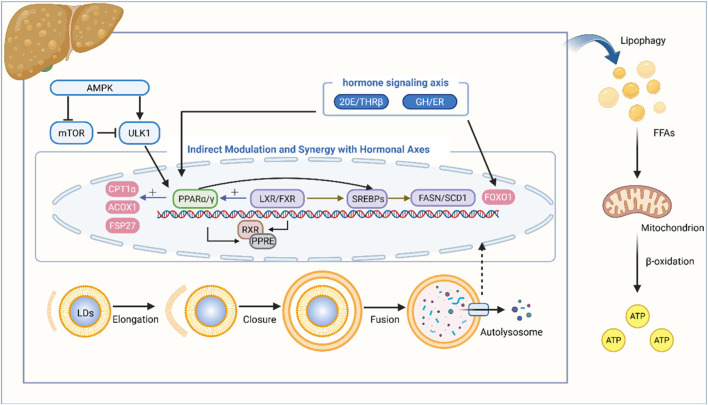
Indirect modulation and synergy with hormonal axes: TCM metabolites targeting receptors to influence lipophagy. During the stage of autophagosome-lysosome docking and fusion, AMPK activates ULK1 and inhibits mTOR. Via the AMPK/mTOR pathway, the PPAR family is activated; these receptors then heterodimerize with RXR to bind to PPREs, thereby upregulating the expression of FA oxidation-related enzymes such as CPT-1A, ACOX1, and FSP27. LXR and FXR, after heterodimerizing with RXR and binding to their respective response elements (LXREs and FXREs), can respectively activate or inhibit SREBPs, thereby upregulating or downregulating the expression of lipogenesis-related genes including FAS and SCD-1. Additionally, FXR induces the expression of PPARα. Hormones such as 20E, THRβ, GH, and ER can modulate the PPAR family and FoxO1 to regulate autophagy. This figure was created with BioRender.com.

In hepatocytes, FXR activation inhibits SREBP-1c expression, a key regulator of hepatic TG synthesis *via* induction of lipogenic enzymes like FASN(Watanabe et al., 2004). This inhibition is mediated through a small heterodimer partner (SHP)-dependent signaling cascade, which antagonizes LXR-mediated SREBP-1c induction by interacting with liver receptor homolog 1 (LRH-1). Beyond suppressing DNL, FXR activation in human cells also induces PPARα and its target genes to enhance FFAs oxidation. Collectively, NRs form interconnected regulatory networks with hormone pathways (e.g., steroid hormones, peptide hormones), synergistically modulating lipophagy through NR-hormone signaling axes (Calkin and Tontonoz, 2012) (Figure 2).

### 5.3 Synergistic hormone signaling axes

Within insects, the steroid hormone 20-hydroxyecdysone (20E) promotes the expression of adipokinetic hormone (AKH) and its receptor (AKHR) *via* its nuclear receptor EcR. 20E elevates hemolymph glucose levels through gluconeogenesis, induces acetylation and nuclear translocation of FoxO1, upregulates PNPLA2 expression in adipocytes to modulate lipid metabolism, enhances lysosomal acid lipase (LIPA) activity, and triggers lipophagy under nutrient deprivation—concomitantly promoting lipase and autophagy gene expression (Li et al., 2025).

Thyroid hormone receptor β (THRβ) is highly expressed in the liver. Upon ligand binding, THRβ undergoes a conformational change, enabling binding to thyroid-responsive elements (TREs) in target gene promoters while recruiting co-activator complexes to directly activate transcription, thereby reducing hepatic TG content (Erion et al., 2007; Davis et al., 2016). Additionally, thyroid hormones indirectly regulate lipid metabolism *via* a THRβ-dependent mechanism by enhancing FoxO1 nuclear translocation, DNA binding, and target gene transcription (Singh et al., 2016). They also upregulate hepatic PPARα signaling to induce CPT-1A expression, thereby increasing FA β-oxidation (Mullur et al., 2014).

Growth hormone (GH) attenuates PPARγ/FSP27 activity and enhances hormone-sensitive lipase (HSL) activity through GHR/JAK2-mediated ERK activation, thereby inhibiting adipogenesis and stimulating lipolysis in human adipocytes (Sharma et al., 2019).

Estrogen enhances hepatic FGF21 expression by activating the Wnt-β-catenin signaling pathway (Badakhshi et al., 2021), thereby promoting hepatic lipophagy. Furthermore, studies demonstrate that estrogen treatment downregulates FA synthesis-related genes such as SCD-1, FASN, ACC, along with PPARγ, and reduces plasma TG levels (Bryzgalova et al., 2006; Palmisano et al., 2016) (Figure 2).

### 5.4 Bioactivity of TCM Constituents

With the rapid advancement of modern molecular biology and pharmacology, research into TCM active metabolites has deepened significantly, emerging as a novel avenue for metabolic disease intervention.

TCM metabolites, such as resveratrol, despite distinct structural origins, can all intervene in MAFLD by precisely regulating the multi-target pathway.

Resveratrol, a natural polyphenol from *Veratrum album* L, specifically upregulates Sestrin2, inhibits the expression of LXRα and SREBP-1c (Jin et al., 2013). It also improves the expression of FXR and SIRT1 expression (Hajighasem et al., 2018). Furthermore, Resveratrol activates the PKA/AMPK/PPARα pathway (Huang et al., 2020), and exerts multi-faceted improvements in NAFLD. Research has demonstrated that resveratrolinduces lipophagy *via* the cAMP-PRKA-AMPK-SIRT1 signaling pathway, partially alleviating MAFLD symptoms (Zhang et al., 2015a).

Herbal metabolites, including Didymin, Nuciferine, and Berberine modulate MAFLD *via* diverse pathways by targeting PPARα-related signaling cascades.

Didymin, a bioactive flavonoid isolated from the peel of *Citrus reticulata* Blanco, has been shown to upregulate the expression of PPARα/β HK1, and ANGPTL4, while downregulating PLIN2 and PTPN1 activity (Fang et al., 2024). Furthermore, didymin promotes lipophagy through activation of SIRT1 and subsequent deacetylation of PGC-1α and FoxO3a, which contributes to the alleviation of MAFLD (Yang et al., 2023).

Nuciferine is an isoquinoline alkaloid extracted from *Nelumbo nucifera* Gaertn, which activates the PPARα/PGC1α signaling pathway in the liver, upregulates the expression of ACOX1, EHHADH, FGF21 (Zhang et al., 2018). It also promotes microtubule-associated protein LC3-II formation in a TFEB-dependent manner, activates the autophagy-lysosome pathway (Du et al., 2022), Synergy accelerates hepatic lipid catabolism, ameliorates steatosis and insulin resistance, and exerts MAFLD-improving effects.

Berberine is an isoquinoline alkaloid extracted from *Coptis chinensis* Franch, It has been shown to downregulate BSCL2 expression, activate the PPARα pathway, and suppress the expression of LD-associated proteins including CIDEA, PLIN4, and PLIN2(Wang H. et al., 2025). Studies have shown that Berberine induces hepatocyte autophagy by activating ERK and inhibiting mTOR pathway (He et al., 2016). These effects collectively reduce LD size, accumulation and mitigate hepatic steatosis.

PPARγ is a key regulator of adipocyte differentiation and glucose metabolism, numerous herbal metabolites have demonstrated robust regulatory potential for this target.

Galangin, a natural flavonoid compound derived from *Alpinia officinarum* Hance, has been shown to upregulate the expression of activated AMPK, suppresse the expression of mTOR, and enhance the expression of Beclin1, ATG3, and LC3-II, thereby promoting the lipophagic activity of cells (Zhang et al., 2020).

Rhein, an anthraquinone compound isolated from *Rheum palmatum* L. By inhibiting the transcriptional activity of LXR, Rhein significantly downregulates the expression of SREBP-1c, FAS, and SCD-1. Concurrently, it reduces the levels of IL-6 and TNF-α. These combined effects mitigate hepatic lipid accumulation and improve insulin resistance (Sheng et al., 2011).

FXR plays a crucial role in regulating BA homeostasis, lipid metabolism, and inflammatory responses. For this NR, numerous herbal metabolites have demonstrated regulatory potential.

Quercetin is widely distributed in edible vegetables, fruits, nuts, and tea. It activates the FXR1/TGR5 signaling pathway and enhances the activities of antioxidant enzymes to improve hepatic lipid metabolism and ameliorates type 2 diabetes-associated MAFLD (Yang et al., 2019). Quercetin also activates AMPK and inhibits mTOR signaling, increases LC3-II expression, enhances hepatocyte lipophagic activity, and activates the P62-Nrf2 antioxidant pathway, reducing hepatic oxidative damage (Katsaros et al., 2024).

Emodin, another anthraquinone compound isolated from *R. palmatum* L, exerts its biological effects primarily by upregulating FXR, activating the IRS-1/PI3K pathway, and inhibiting the gluconeogenic enzymes PEPCK and G6Pase. These combined actions mitigate hepatic lipid accumulation and enhance insulin sensitivity (Shen et al., 2021). Most herbal metabolites can concurrently regulate NRs and lipophagy to treat MAFLD, and potential crosstalk may exist between NRs and lipophagy.

The following experiment directly demonstrated that the active components of TCM exert therapeutic effects through NRs-mediated lipid phagocytosis. further validating the potential of the “TCM–NR–lipophagy” axis.

Dihydromyricetin, a flavonoid compound isolated from *Nekemias grossedentata* (Hand.-Mazz.) J. Wen and Z.L.Nie, activates the AMPK/PGC-1α and PPARα signaling pathways. Concurrently, it upregulates the expression of autophagy-related proteins Beclin1, ATG5, and LC3-II, and promotes autophagosome formation—effects that collectively enhance hepatic lipophagy and reduce lipid accumulation (Yang et al., 2024).

Micheliolide, a sesquiterpene lactone isolated from *Magnolia figo* (Lour.) DC, exerts its hepatoprotective effects by upregulating PPAR-γ expression. Specifically, PPAR-γ upregulation inhibits NF-κB signaling pathway to attenuate hepatic inflammatory responses, while simultaneously activating the AMPK/mTOR pathway to induce lipophagy. These synergistic actions jointly alleviate hepatic steatosis and reduce lipid accumulation (Zhong et al., 2018) (Table 1).

**TABLE 1:** Effect of Chinese medicine monomer *via* NRs and lipophagy on MAFLD.

Name	Chemical structure	Main sources	Targets	Study design	Dosage of administration	Molecular mechanisms	References
Resveratrol	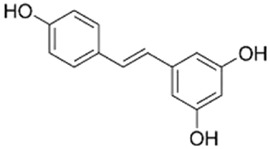	*Veratrum album* L. [Melanthiaceae]	LXRα	C57BL/6J male mice fed HFD HepG2 cells + T0901317	50 mg/kg/d Res for 6 weeks 3,10,30 μM Res for 12 h	Upregulate Sestrin2 to inhibit the LXRα/SREBP-1c pathway	Jin et al. (2013)
			FXR	Wistar male mice fed HFD	25 mg/kg/d Res for 8 weeks	Improvement of SIRT1, LXR and FXR expression	Hajighasem et al. (2018)
			PPARα	SD male mice fed HFD HepG2 cells + PA	100 mg/kg/d Res for 8 weeks 40 μM Res for 24 h	Through the PKA/AMPK/PPARα signaling pathway	Huang et al. (2020)
			lipophagy	129/SvJ mice fed HFD HepG2 cells + PA	Diet containing 0.4% Res for 4 weeks 10,20,40,80 μM Res for 24 h	Induction of lipophagy *via* the cAMP-PRKA-AMPK-SIRT1 pathway	Zhang et al. (2015a)
Didymin	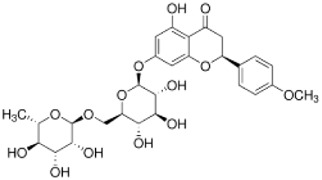	peel of *Citrus reticulata* Blanco [Rutaceae]	PPARs	SD male mice fed HFD LO2 cells + OA	1,2,3 mg/kg/d Didymin for 8 weeks 100 μM Didymin for 48 h	Increase the expression of PPARα and PPARβ to regulate lipid metabolism	Fang et al. (2024)
			lipophagy	C57BL/6J male mice fed HFD AML12 cells + PA	0.8 mg/kg/d Didymin for 3 weeks 50 μM Didymin for 24 h	Enhance the expression of SIRt1, PGC-1α and FoxO3a	Yang et al. (2023)
Nuciferine	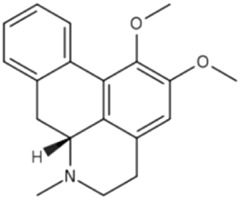	*Nelumbo nucifera* Gaertn. [Nelumbonaceae]	PPARα	C57BL/6J male mice fed HFD	0.06%, 0.12% (w/w) Nuciferine with HFD for 6 weeks	Activation of PPARα/PGC1α signaling pathway	Zhang et al. (2018)
			lipophagy	C57BL/6N male mice fed HFD Hepatocyte-specific TFEB knockout mice + HFD HepG2 cells + PA AML12 cells + PA	0.01%,0.03% Nuciferine per day for 4 weeks 0.03% Nuciferine per day for 4 weeks 100 μM Nuciferine for 12 h 100 μM Nuciferine for 12 h	Activation of the TFEB-mediated autophagy-lysosome pathway enhances lipophagy	Du et al. (2022)
Berberine	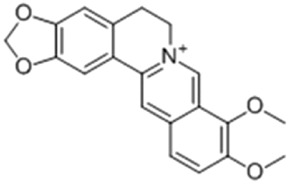	*Coptis chinensis* Franch. [Ranunculaceae]	PPARα	C57BL/6J male mice fed HFD HepG2 cells + OA	150,300 mg/kg/d BBR for 4 weeks 20,30,40 μM BBR for 24 h	Downregulate BSCL2 and activate the PPARα pathway to regulate LD-related proteins	Wang et al. (2025a)
			lipophagy	C57BL/6 male mice fed HFD HFD mice + chloroquine LO2 + FFA LO2 + FFA + bafilomycin A1 LO2 + FFA + SCH772984	50 mg/kg/d BBR for 6 weeks 25 mg/kg/d CQ + 50 mg/kg/d BBR for 6 weeks 0.2,1,5 μM BBR for 24 h 30 nM Baf + 0.2,1,5 μM BBR for 24 h 1 μM SCH + 5 μM BBR for 24 h	Activation of the ERK/mTOR pathway induces lipophagy	He et al. (2016)
Galangin	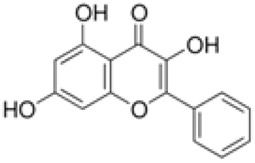	*Alpinia officinarum* Hance [Zingiberaceae]	lipophagy	C57BL/6J male mice fed HFD HepG2 cells + FFA	100 mg/kg/d Galangin for 4 weeks 100 μM Galangin for 12 h	Regulation of the AMPK/mTOR signaling pathway	Zhang et al. (2020)
Rhein	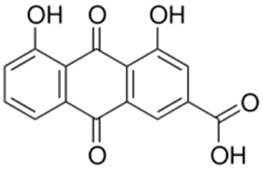	*Rheum palmatum* L. [Polygonaceae]	LXR	C57BL/6J female mice fed HFD Mouse primary T cells + anti-CD3/CD28 antibodies Hepa1-6 hepatoma cells	150 mg/kg/d Rhein for 40 days 25 μM Rhein for 24–48 h 25 μM Rhein + LXR agonist for 48 h	Antagonize LXR and inhibit fat synthesis	Sheng et al. (2011)
Quercetin	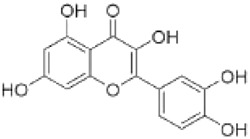	*Styphnolobium japonicum* (L.) Schott [Fabaceae]	FXR	C57BLKS/J background male db/db mice HepG2 cells + HG + FFA	100 mg/kg/d Quercetin for 8 weeks 10,20 μM Quercetin for 24 h	Through antioxidation, anti-inflammatory and activation of FXR1/TGR5 signaling pathways	Yang et al. (2019)
			lipophagy	C57BL/6J male mice fed HFD	10,50 mg/kg/d Quercetin for 4 weeks	Activation of the AMPK/mTOR pathway enhances lipophagocytosis and activates the P62-Nrf2 antioxidant pathway	Katsaros et al. (2024)
Emodin	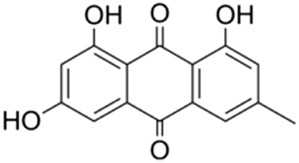	*Rheum palmatum* L. [Polygonaceae]	FXR	C57BL/6 male mice fed HFD FXR^-^/^-^ C57BL/6 male mice Mouse primary hepatocytes + OA+ PA HepG2 cells + OA+ PA	20,40,80 mg/kg/d EMO for 8 weeks 80 mg/kg/d EMO for 8 weeks 12.5,25,50 μM EMO for 24–48 h 25,50 μM EMO for 24 h	Activate the FXR signaling pathway and reduce lipid accumulation in the liver	Shen et al. (2021)
Dihydromyricetin	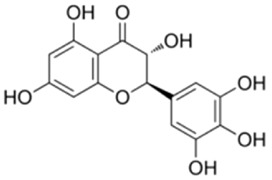	*Nekemias grossedentata* (Hand.-Mazz.) J.Wen and Z.L.Nie [Vitaceae]	PPARα mediated lipophagy	SD male mice fed HFD HepG2 cells + PA	50,100,200 mg/kg/d DHM or 150 mg/kg/d metformin for 6 weeks 5,10,20 μM DHM for 24 h	Activate the AMPK/PGC-1α and PPARα signaling pathways, promoting liver autophagy	Yang et al. (2024)
Micheliolide	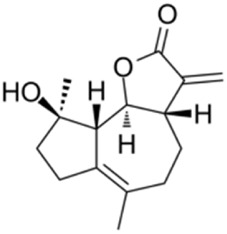	*Magnolia figo* (Lour.) DC. [Magnoliaceae]	PPARγ mediated lipophagy	C57BL/KsJ backround male db/db mice AML12 cells + LM L02 cells + LM	12.5,25,50 mg/kg/d MCL for 16 weeks 2.5,5,10 μM MCL for 24 h	Inhibition of inflammation and promotion of lipophagy mediated by PPARγ	Zhong et al. (2018)

### 5.5 Efficacy of TCM formulas

Chinese herbal compound formulations exhibit the properties of multi-component, multi-target, and multi-pathway actions. Through NR-mediated modulation of lipophagy, these formulations alleviate fatty liver symptoms in experimental animal models, potentially contribute to systematic regulatory improvements in metabolic disorders.

LXRs, as a critical metabolic regulatory hub, represent common targets of numerous effective TCM compound preparations.

QiShenYiQi Pill, a modern TCM compound preparation, targeting the TTC39B-LXRα/β axis, promotes the expression of SR-B1, CYP7A1, and ABCG5, accelerates hepatic cholesterol clearance, reduces hepatic lipid deposition, and ultimately confers anti-atherosclerotic and hepatoprotective effects (Wang et al., 2024). Additionally, the primary active metabolites of QiShenYiQi Pill include Astragaloside, which can respectively promote lipophagy by regulating the Akt/mTOR/TFEB pathway (Zhao et al., 2024).

Yinlan Capsule activates LXRα and FXR, they upregulate the expression of CYP7A1, ABCA1, and LCAT, thereby promoting reverse cholesterol transport, regulating TG metabolism, and inhibiting hepatic lipid synthesis (Chen et al., 2019). The main active metabolites of Yinlan Capsule include Quercetin, which can enhance lipophagy by activating the AMPK/mTOR pathway (Yang et al., 2019).

The Dangfei Liganning Capsule (DFLGN) effectively inhibits DNL of endogenous hepatic lipids by suppressing LXRα activity, collectively improve liver function (Xiaoling et al., 2022). The DFLGN Capsule, which contains Silymarin, regulates the AMPK/mTOR signaling pathway. This regulation induces enhanced lipophagy and reduces lipid deposition (Li et al., 2019).

For the PPAR family, TCM compound preparations also exhibit robust regulatory potential, either by broadly activating PPARs or specifically targeting individual PPAR subtypes to intervene in MAFLD.

Ping-tang Recipe and Jaingpi Qinghua Formula (JPQH) both can regulate the expression of PGC 1α, PPAR α, and PPARγ, enhancing mitochondrial function, promoting FA oxidation, and reducing hepatic lipid deposition (Yang et al., 2012; Xiao et al., 2025). Ping-tang Recipe and JPQH contains Berberine, which activates the ERK/mTOR signaling pathway to induce lipophagy and alleviate hepatic lipid deposition.

The Xiaozhi Formula Si-Ni-San, and ErChen Decoction exert beneficial metabolic effects by targeting specific PPARα subtypes.

Xiaozhi Formula and Si-Ni-San both can upregulate the expression of PPARα, inhibits synthetic genes like FASN and SREBP-1c, reduces hepatic lipid deposition, and effectively improves liver function parameters (Zhang et al., 2023; You et al., 2024). The Xiaozhi Formula contains the active metabolite Rhein, which has been demonstrated to act on lipophagy *via* the AMPK-TFEB pathway (Sheng et al., 2011). Si-Ni-San also contains Paeonol, which activate lipophagy *via* MAPK/mTOR pathway (Deng et al., 2024).

The ErChen Decoction (EC) activates the AMPK signaling pathway, PPARα pathway, and IRS1-Akt-FoxO1 pathway. These actions reduce adipogenesis, promote FA β-oxidation, and improve insulin sensitivity (Wang Y. et al., 2025). The EC Decoction contains Didymin, which enhances the SIRT1-peroxisome PGC-1α-FoxO3a pathway to induce autophagy (Yang et al., 2023).

For the PPARγ nuclear receptor subtype, numerous TCM compound formulas exert effects *via* multi-target mechanisms.

The Huanglian Wendan Decoction (HLWD) regulates the PPARγ/NF-κB signaling pathway, inhibiting pro-inflammatory M1 macrophage polarization in the liver and aorta while promoting anti-inflammatory M2 macrophage polarization. These actions collectively alleviate systemic and local inflammation and improve lipid metabolism (Liu et al., 2025). The HLWD Decoction contains the active ingredient Didymin, which enhances the expression of SIRT1, PGC-1α and FoxO3a to promote lipophagy (Yang et al., 2023).

Qushihuayu Formula, a clinical preparation. It inhibits the MAPK and NF-κB signaling pathway, promotes PPARγ nuclear translocation, thereby improving lipid metabolism and inflammation (Lan et al., 2021). Its main components include Quercetin, modulating lipophagy *via* the AMPK/mTOR pathway to improve MAFLD (Yang et al., 2019).

For the FXR nuclear receptor, Yin-chen Wu-ling Powder modulates MAFLD *via* multi-faceted actions of its active components.

Yin-chen Wu-ling Powder, a classic herbal formula. On one hand, it binds to FXR to regulate BA metabolic balance; on the other hand, it inhibits activation of the Akt/P38 MAPK signaling pathway, reducing IL-1β, IL-6, and TNF-α release and inflammatory cell infiltration. These dual actions reduce liver injury and inflammation caused by bile stasis, conferring hepatoprotective effects (You et al., 2023). Additionally, components like Berberine, Atractylenolide I, alisol B 23-acetate have been identified as potential FXR agonists, further elucidating the formula’s mechanism of action (Zou et al., 2023). Yin-chen Wu-ling Powder and Shenling Baizhu Powder share herbs like *Atractylodes macrocephala* Koidz. [Asteraceae], which regulate the SIRT1 and Akt to mediate lipophagy, improve lipid deposition. These effects Yin-chen Wu-ling Powder may also exhibit (Pan et al., 2024).

The aforementioned animal studies provide valuable empirical evidence for the efficacy of TCM compound formulas in treating MAFLD and partial mechanistic insights (Table 2).

**TABLE 2:** TCM compound acts on MAFLD through various pathways.

Name	Main medicinal herbs	Targets	Study design	Dosage of administration	Molecular mechanisms	References
QiShenYiQi pill	*Astragalus membranaceus, Salvia miltiorrhiza,* *Panax notoginseng,* *Dalbergia odorifera*	LXRs	ApoE^−/−^ male mice fed HFD BRL3A cells + Ttc39b siRNA interference	0.3,1.2 g/kg/d QSYQ or 10 mg/kg/d GW3965 for 8 weeks QSYQ-containing serum +1 mmol/L FFA for 24 h	Inhibition of TTC39B stabilizes LXRs, thereby activating the hepatic cholesterol reverse transport pathway	Wang et al. (2024)
Yinlan Capsule	Leaf of *Ginkgo biloba, Gynostemma pentaphyllum*	LXRα	Wistar mice fed HFD	36,72,144 mg/kg/d YL Capsule for 30 days	Activating LXRα promotes the conversion of TC to BAs and the reverse transport of TC in the periphery	Chen et al. (2019)
Dangfei Liganning Capsule	*Silybum marianum* (L.) Gaertn. [Asteraceae], *Swertia bimaculata* (Siebold & Zucc.) Hook.f. and Thomson ex C.B.Clarke [Gentianaceae]	LXRα	SD male mice fed HFD	0.0675,0.135,0.27 g/kg/d DFLGN Capsule or 0.123 g/kg/d Essentiale for weeks	Block the LXRα-SREBP-1-FAS signaling pathway	Xiaoling et al. (2022)
Ping-tang Recipe	*Alisma plantago-aquatica subsp. orientale,* *Atractylodes macrocephala,* *Rheum palmatum,* *Crataegus pinnatifida*	PPARs	SD male mice fed HFD	0.42,0.84 g/kg/d PTR for 8 weeks	Activate AMPK, upregulate the expression of PPARs and inhibit SREBP-1c	Yang et al. (2012)
Jaingpi Qinghua Formula	root of *Pueraria montana* (Lour.) Merr. [Fabaceae], *Astragalus membranaceus* (Fisch.) Bunge (Fabaceae), *Codonopsis pilosula* (Franch.) Nannf	PPARs	C57BL/6 male mice fed HFD	0.1 mL/10 g/d JPQHF or Metformin for 6 weeks	Increase PGC1α, PPARα, and CPT1A; decrease PPARγ	Xiao et al. (2025)
Xiaozhi Formula	*Nelumbo nucifera,* *Trichosanthes kirilowii,* *Persicaria perfoliate,* *Salvia miltiorrhiza*	PPARα	C57BL/6J male mice fed HFD	2.835,5.67 g/kg/d XZF for 8 weeks	Activate the AMPK/PPARα pathway, promoting lipophagy and inhibiting synthesis	You et al. (2024)
Si-Ni-San	*Cynanchum otophyllum,* *Bupleurum falcatum,* fruit of *Citrus × aurantium,* *Glycyrrhiza uralensis*	PPARα	SD male mice fed HFHCD HepG2 cells + PA	2,4 g/kg/d SNS or 150 mg/kg/d Metformin for 4 weeks 10% SNS-containing serum or AMPK inhibitor treatment	Activate the AMPK/SIRT1 signaling pathway and promote the expression of PPARα	Zhang et al. (2023)
ErChen Decoction	peel of *Citrus reticulata,* *Glycyrrhiza uralensis*	PPARα	HepG2 cells + OA+ PA HepG2 cells + insulin	0.25,0.5,1 mg/mL ECD extract for 48 h 0.125,0.5 mg/mL ECD extract for 24 h	Activate the AMPK and PPARα signaling pathways	Wang et al. (2025b)
Huanglian Wendan Decoction	*Coptis chinensis,* peel of *Citrus reticulata*	PPARγ	ApoE^-^/^-^ male mice fed HFHCD	7.05,14.09,28.18 g/kg/d HLWDD or 5.28 mg/kg/d Pioglitazone for 7 weeks	Regulate the PPARγ/NF-κB signaling pathway	Liu et al. (2025)
Qushihuayu Formula	*Gardenia jasminoides,* *Achyranthes bidentata*	PPARγ	Wistar male mice fed MCDD	0.29,0.57,1.14 g/kg/d QSHYF for 8 weeks	Inhibit the phosphorylation of MAPK and promote the nuclear translocation of PPARγ	Lan et al. (2021)
Yin-chen Wu-ling Powder	*Alisma plantago-aquatica subsp. orientale,* *Atractylodes macrocephala*	FXR	Mdr2^-^/^-^ male mice	14.99 g/kg/d YCWLP or 91 mg/kg/d UDCA for 60 days	Combined with FXR, it regulates the balance of BA metabolism; inhibits the Akt/P38MAPK signaling pathway	You et al. (2023)
​	​	FXR	C57BL/6 male mice fed ANIT HEK293T cells	1.5,3 g/kg/d YCWLP for 2 weeks 15,30 μM YCWLP components for 24 h	Activate FXR and regulate BA homeostasis	Zou et al. (2023)

## 6 Challenges and future perspectives

Although current research has confirmed the potential value of TCM in the prevention and treatment of MAFLD, existing mechanistic investigations primarily focus on the regulation of lipid metabolism by TCM *via* NRs—while lipophagy, the equally critical process of autophagic lipid degradation, has received insufficient attention. Preliminary evidence suggests that TCM interventions may target both NRs and lipophagy simultaneously. Therefore, future research urgently needs to move beyond the single-pathway perspective and focus on exploring how NRs precisely regulate lipophagy activity under TCM intervention, thereby constructing a multi-dimensional regulatory axis centered on the “TCM-NRs-lipophagy” framework. In-depth elucidation of the molecular mechanisms underlying this axis will not only fill a critical gap in the current understanding of MAFLD pathogenesis but also provide a robust theoretical basis for developing innovative prevention and treatment strategies that leverage the multi-targeted and holistic regulatory properties of TCM.

During the modernization of TCM research, several challenges persist, particularly in the context of developing TCM-based interventions targeting the NR-lipophagy axis for MAFLD. TCM compound formulas typically contain numerous active metabolites with complex and diverse compositional profiles, and many active metabolites that may modulate the NR-lipophagy axis remain challenging to characterize precisely (Dong et al., 2019). Additionally, TCM metabolites with potential regulatory effects on this axis face inherent challenges including low bioavailability, poor aqueous solubility, and rapid metabolic clearance—limitations that hinder their ability to reach liver targets and exert sustained effects on NRs or lipophagy (Wang et al., 2021; Mirza et al., 2023; Yang et al., 2025). Concurrently, the lack of standardized quality control protocols for TCM herbal medicines presents substantial obstacles to elucidate active metabolites and their specific roles in regulating the NR-lipophagy axis (Zhou et al., 2019). To ensure quality control of TCM preparations designed for MAFLD, HPLC fingerprinting can be employed to analyze the variation trends of multiple indicator components, especially those linked to NR activation or lipophagy modulation. This approach effectively guarantees batch-to-batch consistency of the products, a critical prerequisite for reproducible modulation of the NR-lipophagy axis (Wang et al., 2007). By developing liver-targeted delivery systems, formulating nanoscale TCM preparations, and leveraging microbial biotransformation, the bioavailability of TCM components can be enhanced, associated adverse reactions reduced, and the aqueous solubility and physicochemical properties of TCM improved—all of which are essential for optimizing the modulation of the NR-lipophagy axis (Wang et al., 2021; Wei et al., 2022; Athanasopoulou et al., 2023). In clinical trial design for TCM-based MAFLD therapies, in addition to conventional efficacy endpoints, biomarkers associated with the NR and lipophagy-mediated mechanism of action should be incorporated to confirm the drug’s multi-target mechanism of action in clinical settings.

Driven by advances in modern translational medicine, the integration of TCM with cutting-edge computational technologies has positioned the development of novel MAFLD-targeted therapeutics as a research frontier. Integrating TCM’s inherent multi-component, multi-target properties with modern computational drug design methodologies hold promise for developing next-generation NR dual modulators that simultaneously regulate key NRs and lipophagy-related pathways, thereby providing innovative strategies for the precision treatment of MAFLD and accelerating TCM modernization.

## Glossary

MAFLD: Metabolic-associated fatty liver disease
TGs: Triglycerides
LDs: Lipid droplets
FFAs: Free fatty acids
NRs: Nuclear receptors
PPARs: Peroxisome proliferator-activated receptors
FXR: Farnesoid X receptor
LXR: Liver X receptor
mTORC1: mammalian Target of Rapamycin Complex 1
AMPK: AMP-activated protein kinase
ULK1: Unc-51 like autophagy activating kinase 1
PI3K: Phosphatidylinositol 3-kinase
ULK2: Unc-51 like autophagy activating kinase 2
ATG13: Autophagy-related protein 13
ATG101: Autophagy-related protein 101
FIP200: FAK-family interacting protein of 200 kDa
VPS34: Vacuolar protein sorting 34
ATG14L: Autophagy-related protein 14-like
AMBRA1: Activating molecule in BECN1-regulated autophagy protein 1
PI3P: Phosphatidylinositol 3-phosphate
WIPIs: WD repeat domain phosphoinositide-interacting proteins
DFCP1: Double FYVE-containing protein 1
ER: Endoplasmic reticulum
CETP: Cholesteryl ester transfer protein
LC3-II: Microtubule-associated protein 1A/1B-light chain 3-II
P62: Sequestosome-1
OPTN: Optineurin
NBR1: Neighbor of BRCA1 gene 1
TFEB: Transcription factor EB
CLEAR: Coordinated lysosomal expression and regulation
TFE3: Transcription factor E3
FoxO1: Forkhead box protein O1
CCND1: Cyclin D1
DNM2: Dynamin 2
HOPS: Homotypic fusion and protein sorting
LAL: Lysosomal acid lipa
DNL: *De novo* lipogenesis
IRGM: Immune-related guanosine triphosphatase M protein
SREBP: Sterol-regulatory element-binding protein
GMNT: Glycine N-methyltransferase
TSC2: Tuberous sclerosis protein 2
Akt: Protein kinase B
FGF21: Fibroblast growth factor 21
NF-κB: Nuclear factor kappa-B
BA: Bile acids
SHP: Small heterodimer partner
NASH: Non-alcoholic steatohepatitis
FASN: Fatty acid synthase
NLRP3: NOD-like receptor thermal protein domain associated protein 3
CCL-2: C-C motif ligand 2
ECM: Extracellular matrix
TGF-β1: Transforming growth factor-β 1
LPL: Lipoprteinlipase
APO: Apolipoprotein
CPT: Carnitine palmityl transferase
OCA: Obeticholic acid
LDL: Low-density lipoprotein
HDL: High density lipoprotein
BBR: Berberine
ERR: Estrogen-related receptor
TZD: Thiazolidinedione
VDR: Vitamin D receptor
PPRE: Peroxisome proliferator response element
ACOX1: Peroxisomal acyl-coenzyme A oxidase 1
FSP27: Fat-specific protein 27
20E: 20-Hydroxyecdysone
AKH: Adipokinetic hormone
THRβ: Thyroid hormone receptor β
TRE: Thyroid-responsive element
GH: Growthhormone
SCD: Stearoyl-CoA desaturase
ACC: Acetyl-CoA carboxylase
ANIT: Alpha-naphthyl isothiocyanate
Nrf2: Nuclearrespiratoty factor 2
TNF: Tumor necrosis factor
INF: Interferon
IL-6: Interleukin- 6
AST: Aspartate aminotransferase
ALT: Alanine aminotransferase
OA: Oleic Acid
PA: Palmitic acid
HFD: High-fat diet
MCD: Methionine and choline deficient
HFHCD: High-fat high-cholesterol diet
DFLGN: Dang-Fei-Li-Gan-Ning
JPQH: Jian-Pi-Qing-Hua
HLWD: Huang-Lian-Wen-Dan
EC: Er-Chen
YCWL: Yin-Chen-Wu-Ling
